# In Situ Mo(Si,Al)_2_-Based Composite through Selective Laser Melting of a MoSi_2_-30 wt.% AlSi10Mg Mixture

**DOI:** 10.3390/ma13173720

**Published:** 2020-08-23

**Authors:** Tatevik Minasyan, Sofiya Aydinyan, Ehsan Toyserkani, Irina Hussainova

**Affiliations:** 1 Department of Mechanical and Industrial Engineering, Tallinn University of Technology, Ehitajate 5, 19086 Tallinn, Estonia; sofiya.aydinyan@taltech.ee; 2 Multi-Scale Additive Manufacturing Laboratory, Department of Mechanical and Mechatronics Engineering, University of Waterloo, 200 University Ave., West Waterloo, ON N2L 3G1, Canada; ehsan.toyserkani@uwaterloo.ca

**Keywords:** selective laser melting, molybdenum disilicide, composite, aluminum alloy, computed tomography, surface roughness

## Abstract

The laser power bed fusion approach has been successfully employed to manufacture Mo(Si,Al)_2_-based composites through the selective laser melting of a MoSi_2_-30 wt.% AlSi10Mg mixture for high-temperature structural applications. Composites were manufactured by leveraging the in situ reaction of the components during printing at 150–300 W laser power, 500–1000 mm·s^−1^ laser scanning speed, and 100–134 J·mm^−3^ volumetric energy density. Microcomputed tomography scans indicated a negligible induced porosity throughout the specimens. The fully dense Mo(Si_1-x_,Al_x_)_2_-based composites, with hardness exceeding 545 HV1 and low roughness for both the top (horizontal) and side (vertical) surfaces, demonstrated that laser-based additive manufacturing can be exploited to create unique structures containing hexagonal Mo(Si_0.67_Al_0.33_)_2_.

## 1. Introduction

Molybdenum disilicide MoSi_2_ is regarded as a promising material for a wide variety of industrial applications due to its combination of outstanding properties, such as its high melting point (2293 K), moderate density (6.24 g·cm^−3^), excellent intermediate- and high-temperature oxidation resistance, and metallic-like high electrical and thermal conductivity [1]. Exploitation of these properties opens up the prospect for the development of composites with tailored mechanical and physical behaviours. Aluminum is one of the most favorable alloying additions for MoSi_2_, which may enhance the high-temperature oxidation resistance, fracture toughness, creep resistance, and low-temperature ductility of MoSi_2_, along with introducing a more metallic character to the compound [2].

The main processing routes explored so far for preparation of Mo(Si,Al)_2_ are arc melting [3,4,5,6], self-propagating high-temperature synthesis (SHS) [7,8,9,10], hot pressing [11], spark plasma sintering (SPS) [12], and some others [13], as specified in Table 1.

Different Si/Al ratios in Mo(Si_1-x_,Al_x_)_2_ yield the formation and development of C40 hexagonal and C54 orthorhombic structures. The duplex C40/C54 phases appear when x = 0.5, while monophasic C54, often designated as Mo_10_Si_7_Al_13_, is established at x = 0.6, as described in [11]. In [9], a similar trend of C54 phase formation was reported. It was also found that at 17.86 wt.% Al addition, the C54 phase with an Al_4_Mo_3_Si_2_ composition is developed [10]. A higher percentage of Al does not promote the development of monophasic C54, since the segregation of Mo suppresses the combustion synthesis. The formation of C40 Mo(Al_0.5_,Si_0.5_)_2_ and the orthorhombic C54 MoAl_1.3_Si_0.7_, as a result of the arc-melted mixture of 33.3 at.% Mo, 44.2 at.% Si, and 22.5 at.% Al, is reported in [4]. The Al-rich side of the Mo(Si,Al)_2_ composition ends at the Al_8_Mo_3_ phase, as described in [14].

The main difficulties associated with the processing of MoSi_2_-AlSi10Mg composites are the restrictive stoichiometry limits, high melting point, and low-temperature brittleness. Application of additive manufacturing (AM) techniques for the MoSi_2_-Al system has not been explored yet. AM through selective laser melting (SLM) provides a wide range of new opportunities for the processing and fabrication of various products with complex constitutional designs and shapes, which are impossible to achieve via any conventional method.

In this paper, an attempt is made to produce Mo(Si,Al)_2_-based composites in situ, exploiting the laser power bed fusion of MoSi_2_ and AlSi10Mg alloy powders and taking into consideration the successful SLM of MoSi_2_ [15,16] and AlSi10Mg [17]. In this context, we demonstrate for the first time the reactive selective laser melting of MoSi_2_-30 wt.% AlSi10Mg, which results in fully dense bulks of good quality. This paper discusses the material structure and hardness; however, the high-temperature mechanical properties and oxidation are out of this paper’s scope.

## 2. Experiments

### 2.1. Powder Preparation and Characterization

For SLM feedstock preparation, combustion-synthesized MoSi_2_ powder (99% purity) was mixed with 30 wt.% gas-atomized AlSi10Mg (SLM, Solutions, purity >99%, 15–63 µm) for 3 h using mechanical rotation (20 rpm). The particle size and sphericity analyses of the MoSi_2_, AlSi10Mg, and MoSi_2_-30 wt.% AlSi10Mg powders were carried out in a CAMSIZER X2 device (MICROTRAC MRB, Haan, Germany), adopting dynamic image analysis (ISO 13322-2 [18]) as the measuring principle using 10 mL of powder. Sphericity is determined as the square of the circularity of a powder particle using the 4$\sqrt{A}$·P^−2^ equation, where P is the measured perimeter of the particle and A is the computed area from the particle projection. The FT4 Powder Rheometer (Freeman Technology, Tewkesbury, UK) was used to determine the basic flowability energy (BFE) of powders and mixture. To define the resistance of a powder to flow, 8 test cycles were run. During each test, the precision blade rotated downwards (at a speed of −100 mm·s^−1^) and upwards through the fixed volume of powder to create a flow pattern. The BFE is calculated with the following equation: BFE = E_test8_, where E_test8_ is the energy recorded during downward rotation of the blade for the 8th test. The stability index (SI) is calculated as follows: SI = E_test8/_E_test1._ The packing density of the mixture was measured by GranuPack (GranuTools, Awans, Belgium). The actual value for the average bulk density at the end of the trials (ρ[n]) was measured and the bulk density value for ρ[∞] was extrapolated.

Differential scanning calorimetry (DSC) analysis was performed to analyze the thermal behavior of MoSi_2_-30 wt.% AlSi10Mg powder (70 mg) using a NETZSCH-STA 449 F1 Jupiter (NETZSCH-Gerätebau GmbH, Selb, Germany) thermal analyzer from 25 °C to 1450 °C, with a heating rate of 20 °C·min^−1^ in an argon atmosphere.

### 2.2. Selective Laser Melting

Consolidation of the MoSi_2_-30 wt.% AlSi10Mg mixture by selective laser melting (SLM) was carried out using a Renishaw AM400 apparatus (Wotton-under-Edge, Gloucestershire, UK), which employs a high-power, continuous-wave, and a laser, modulated to work as a pulse laser. The device is equipped with an Ytterbium laser with a maximum power of 400 W and a wavelength of 1.07µm. Cylindrical samples with dimensions of ∅7 mm × 7 mm were built. The meander scan strategy was used, whereby scan patterns rotate by 67° after each printed layer. The whole manufacturing process was carried out inside a chamber containing a precisely controlled atmosphere of argon at an oxygen level below 500 ppm. The hatching distance (h) and laser spot size were set as 85 µm and 90 µm, respectively. The laser power (P) was in the range of 150–300 W and the scanning speed (ν) was in the range of in 500–1000 mm·s^−1^ range. The layer thickness (d) was chosen as 35 µm. The process parameters for preparation of the three samples are listed in Table 2.

The laser volumetric energy density (LED) and build rate were calculated according to E = P/νhd and BR = *νhd*, respectively.

### 2.3. Bulk Characterization

The density of printed parts was measured by Archimedes’ principle (Mettler Toledo ME204, Greifensee, Switzerland) and by calculating the dimensions (digital Vernier caliper of 0.01 mm accuracy, Digital Caliper, KS Tools Werkzeuge-Maschinen GmbH, Heusenstamm, Germany) and weight (Eltra 84 analytical balance, 0.1 mg accuracy, Haan, Germany) of the samples. The size, morphology, and alignment of the pores in the SLM-fabricated samples were inspected via X-ray computed tomography (CT) (ZEISS Xradia 520 Versa 3D X-ray microscope Oberkochen, Germany). The surface roughness analysis was performed using a Keyence VK-X250 profile analyzing confocal laser microscope (Keyence Corporation, Osaka, Japan). On each studied surface, 4 areas with dimensions of 500 μm × 700 μm were scanned using a 20× lens. The maximum height (Sz) and arithmetical mean height (Sa) were averaged for 4 measurements.

Samples were ground and polished by conventional metallographic methods to a 0.5 μm diamond polish and to a finer 0.1 μm finish using a nylon disc and polishing suspension. The morphology and microstructure of the powders and printed bulks were examined by TESCAN VEGA3 (Brno, Czech Republic) and HR-SEM Zeiss Merlin scanning electron microscope (SEM, ZEISS, Oberkochen, Germany) equipped with an EDS detector (Bruker EDX-XFlash6/30, Billerica, MA, USA ). Phase compositions were analyzed with the help of an X-ray diffractometer (Siemens/Bruker D5000 X-ray Powder Diffraction (XRD) System, Billerica, MA, USA) with CuKα radiation in the 2θ range of 20° to 80°. The concentrations of compounds and elements were estimated via the Rietveld refinement method. The Vickers hardness values were measured on the polished surfaces of the printed specimens using a tester (Indentec 5030 SKV, Stourbridge, West Midlands, UK) at a load of 9.8 N, applied over 10 s for 10 indents.

## 3. Results and Discussion

### 3.1. Powders

**Figure 1:**
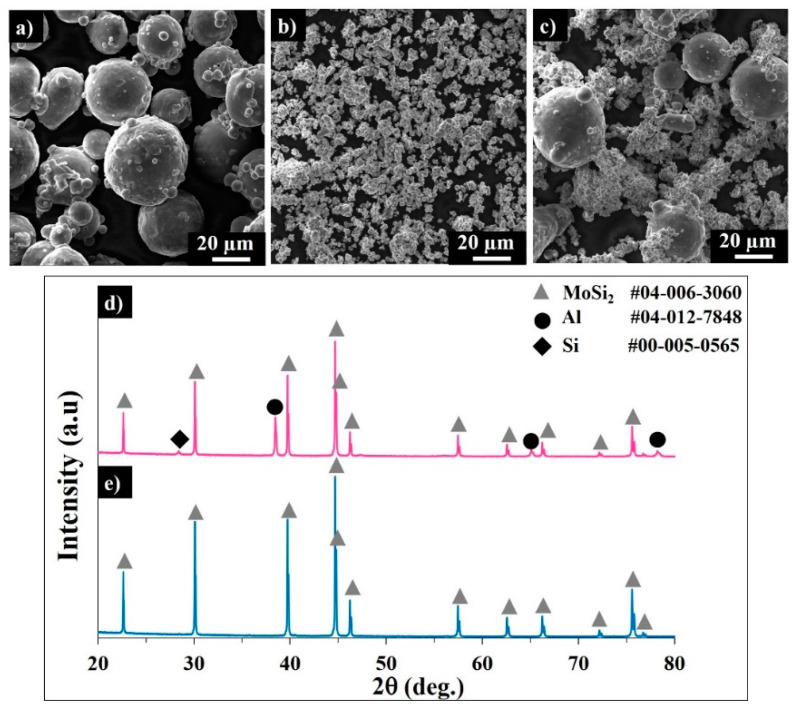
SEM images of the AlSi10Mg alloy (**a**) MoSi_2_ and (**b**) MoSi_2_-30 wt.% AlSi10Mg powder mixture (**c**). Corresponding XRD patterns of the MoSi_2_-30 wt.% AlSi10Mg (**d**) and MoSi_2_ (**e**) powders.

Powder flowability is one of the most influencing parameters affecting the powder bed density, and therefore affecting the produced item quality, including the density and roughness. [15,17]. The powder particle shape, size, and distribution are of primary importance for sintering kinetics and powder bed formation [17,19]. The SEM image of the gas-atomized AlSi10Mg alloy powder is shown in Figure 1a. As shown in Table 3, the median diameter (D50) of AlSi10Mg was evaluated as ~40 µm. The powder of MoSi_2_ consists of agglomerates of fine (1–5 µm) particles (Figure 1b), with D50 < 19 µm (Table 3). The SEM image of the MoSi_2_-30 wt.% AlSi10Mg powder mixture clearly demonstrates an apparent particle size difference between constituents. Fine MoSi_2_ particles can serve as nucleation centers during cooling and provide homogeneous solidification after laser scanning.

Usually, the spherical particles of narrow size distribution exhibit better flowability compared to the angularly shaped powders. The sphericity of the MoSi_2_-30 wt.% AlSi10Mg mixture gradually decreased from ~0.9 to 0.79 in a particle size range of 3–55 µm, conditioned by the non-spherical shape of the MoSi_2_ agglomerates.

The basic flowability energy values of AlSi10Mg, MoSi_2_-30 wt.% AlSi10Mg, and MoSi_2_ were not significantly different, being ~206, 252, and 307 mJ, respectively (Table 4). Therefore, the flowability of the mixture can be considered as fair and applicable for SLM processing.

**Figure 2:**
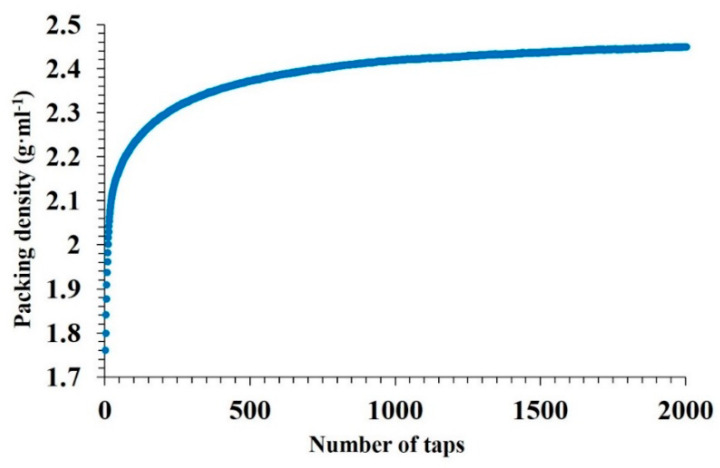
Average packing density of the MoSi_2_-30 wt.% AlSi10Mg powder as a function of stress.

The bulk density of the powders is another important characteristic when choosing the powder feedstock for SLM. Figure 2 shows the packing density of the MoSi_2_-30 wt.% AlSi10Mg powder mixture. The initial bulk density of the powder was 1.76 ± 0.012g·mL^−1^; after 2000 taps, the bulk density increased up to 2.45 ± 0.048 g·mL^−1^ and the packing density was extrapolated to be 2.62 ± 0.064 g·mL^−1^.

Small particles of MoSi_2_ can fill voids between the large particles of AlSi10Mg and increase the packing density.

### 3.2. Bulks

**Figure 3:**
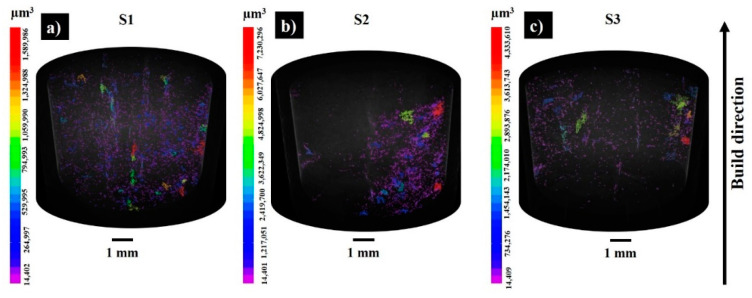
The 3D visualization of the porosity results in as-built samples S1 (**a**), S2 (**b**), and S3(**c**) (pores are labelled by volume (μm^3^)).

**Figure 4:**
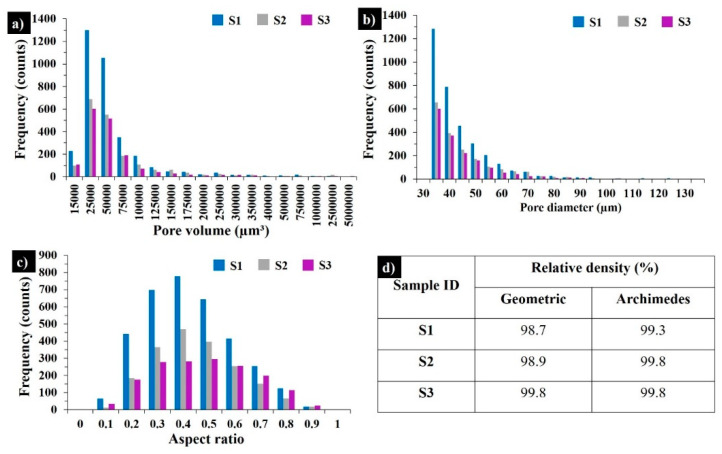
X-ray CT results in as-built samples S1, S2, and S3: pore size distributions (**a**), pore diameter distributions (**b**), aspect ratios of the pores (**c**), and relative geometric and Archimedes density results (**d**).

Figure 3 demonstrates the 3D visualization of porosity in samples S1–S3. The porosity level calculated by part and pore voxels is less than 1% when supposing that the relative density of the bulks is ≥99%. Figure 4 displays X-ray CT analysis of the pore distribution and its aspect ratio. The samples S1 and S3 are printed with the same applied energy density (100.8 J·mm^−3^), but at different scanning speeds and laser powers. The sample S3 possesses the lowest number of pores (Figure 3c and Figure 4), pointing to the effect of the laser power on the decreased porosity; however, further SEM analyses revealed the susceptibility to cracking of the materials printed with a high laser power (300 W). For samples S1 and S2, the maximum frequency was observed for the pores with an aspect ratio of 0.4, while for S3 most of the pores had an aspect ratio of 0.5 (Figure 4c). For all three samples, the majority of the pores measured were of 30 – 35 µm. The relative geometric and Archimedes density results coincided with the CT scanning results (Figure 4d).

**Figure 5:**
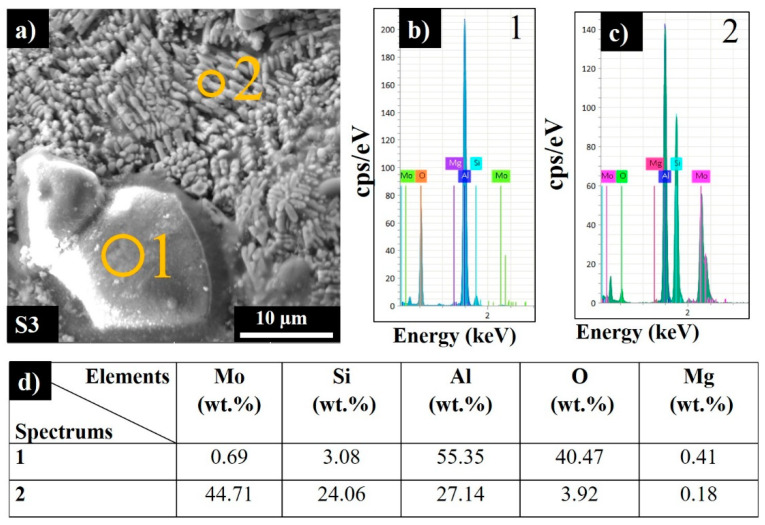
Side surface SEM image (**a**), EDS spectra (**b**,**c**), and corresponding elemental analysis results for sample S3 (**d**).

The average surface roughness (Sa) and the maximum height (Sz) of the top and side surfaces of the printed samples are listed in Table 5. A two-fold increase in the scanning speed results in a moderate increase in Sa and a dramatic increase in the maximum heights of irregularities for both top and side (vertical) surfaces. S2 produced the highest LED value but a moderate scan speed and possessed the smoothest top surface, which was conditioned by sufficient melting and sintering of the consecutive powder layers. The quality of the side surfaces was slightly inferior compared to the top surface. Particles on the sides were primarily formed by the partial melting at the build surface. For S3, a high applied LED value combined with a high build rate resulted in adhesion of the powder particles from the heat-affected zones onto the side surface of the piece, resulting in high surface roughness. The partially sintered particles detected on the top surface might be due to the blowing of metal particles into the laser-melted zones by the gas flow in the build chamber or from the powder bed due to vibration movement of the wiper during processing. Moreover, aluminum oxide can be formed due to oxidation of AlSi10Mg by the trace oxygen in the inert gas or by the inherent alumina layer on the powder surface. According to the EDS analysis, the adhered particles were examined and determined to be aluminum oxide (Figure 5, Spectrum 1).

Spectrum 2 (Figure 5c) shows that the chosen section was composed of 44.71 wt.% Mo 24.06 wt.% Si and 27.14 wt.% Al, along with 3.92 wt.% oxygen.

### 3.3. Microstructural Analysis

**Figure 6:**
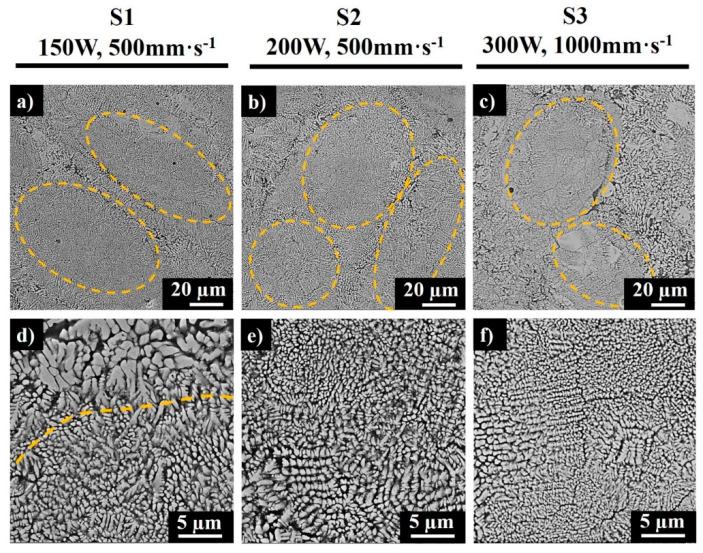
SEM images of polished top surfaces of samples S1 (**a**,**d**), S2 (**b**,**e**), and S3 (**c**,**f**).

The SEM images of the polished top surfaces of samples S1, S2, and S3 are depicted in Figure 6. The marked elliptical regions in Figure 6a–c demonstrate the core of the melt pools. The solidification process affects the crystallization in different regions due to various temperature gradients and heat flux directions, depending on the laser power and scanning speed. The melt pool dimensions depend on the applied laser power, LED, and temperature. There was a larger heat flux in the center of the melt pool due to Gaussian intensity of the single mode laser beam; hence, the surface tension varied between the center and outer edges, resulting in multifarious microstructures along the sample. Importantly, controlling the volume of the melt by increasing the scan speed was demonstrated to reduce the overlap and cause insufficient melting (Figure 6c).

The cores consisted of fine submicron- to micron-sized grains (Figure 6d–f), while at the border of each elliptical section, the coarser columnar dendrites were observed (Figure 6d,e). This phenomenon is caused by the cooling rate and temperature difference in the center of the melt pool and at the border, as the melt pool periphery is exposed to a longer laser exposure during overlapping of adjacent scan tracks. From the microstructure of S3 produced at the scan speed of 1000mm·s^−1^ (Figure 6c), a higher inhomogeneity of the morphological texture was detected as compared to samples S1 and S2. The bright regions in Figure 6 represent sintered MoSi_2_, while the dark grey regions represent the segregated Al-rich phase that developed due to the incomplete reaction between MoSi_2_ and Al at high scanning speeds. The light grey phase characterizes the Mo-Si-Al phase (which according to further XRD analysis was found to be the Mo_3_(Al_2_Si_4_) phase). A high scanning speed decreases the interaction time between a laser and a material, thus decreasing the solidification time and inducing a high temperature gradient [20]; therefore, a higher cooling rate at the interface results in a finer microstructure, as seen from Figure 6f.

**Figure 7:**
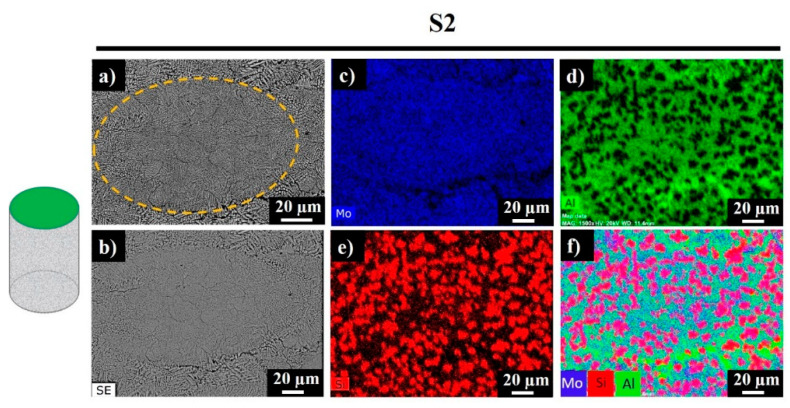
Top surface backscattered electron (BSE) image (**a**), secondary electron (SE) image (**b**), and EDS mapping results (**c**–**f**) for sample S2.

Figure 7 demonstrates the backscattered electron (BSE) and secondary electron (SE) images of S2, along with corresponding elemental and mixed EDS maps.

**Figure 8:**
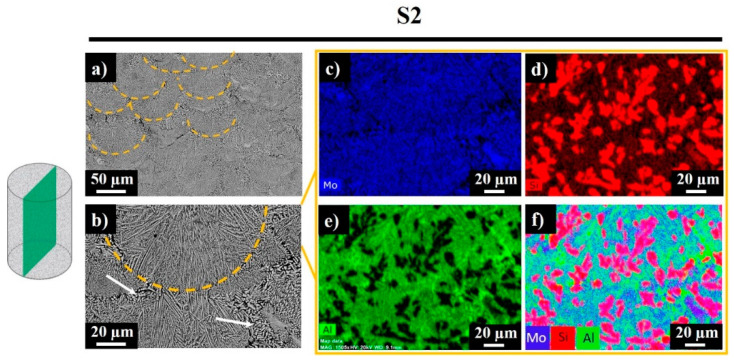
Top surface SEM images (**a**,**b**) and EDS mapping results for sample S2 (**c**–**f**).

Signals of Mo were recorded everywhere expect the darkest regions, which correspond to the Al-rich phase (Figure 7d, bright green regions). Silicon was also observed in the studied area, while the bright red sections revealed the existence of replaced free Si, where no Al was detected. Accordingly, the sample was composed of Mo-Si-Al-, Al-, Al-, and Si-rich phases. Figure 8 shows the polished side fracture of sample S2 and the corresponding EDS maps. The yellow dashed regions (Figure 8a,b) represent the melt pools. The core of the melt pool was composed of fine elongated columnar dendrites with secondary arms grown parallel to the build direction, whereas the edges consisted of the coarser columnar dendrites (marked with white arrows) with occasional secondary branching due to overlap with the neighboring melt pools (Figure 8b).

The EDS maps corresponding to the SEM image in Figure 8b reveal a similar composition as for the top surface of the sample. Most of the studied area was composed of the Mo-Si-Al-containing phase. The dark regions in Figure 8b reveal the absence of Mo and the presence of the Al-rich phase. The black regions in the Al green map and the bright red regions in the Si map disclose the partial substitution of Si by Al.

**Figure 9:**
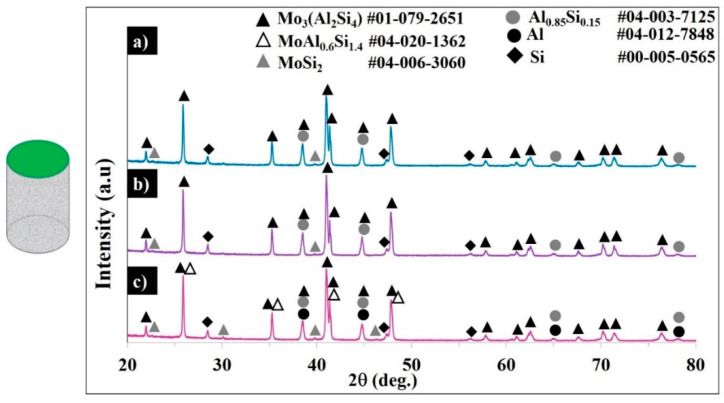
XRD patterns of samples S1 (**a**), S2 (**b**), and S3 (**c**).

Figure 9 displays the XRD patterns and Table 6 shows the phases and elemental compositions of the samples according to the Rietveld refinement method.

The diffractograms of sample S3 confirm the presence of tetragonal MoSi_2_, face-centered cubic Al, the substituted face-centered Si, the coexistence of the in-situ-formed hexagonal Mo_3_(Al_2_Si_4_)/MoAl_0.6_Si_1.4_ phases (corresponds to Mo(Si_1-x_,Al_x_)_2,_ x = 0.3–0.33) composition), and the Si-saturated Al-rich Al_0.85_Si_0.15_ phase.

The patterns for samples S1 and S2 evidenced the complete transformation of MoSi_2_ to C40 Mo_3_(Al_2_Si_4_), with weak peaks of unreacted C11b, negligible MoAl_0.6_SiSi_1.4_, and with Al_0.85_Si_0.15_ being formed. For the applied high scanning speed, apparently the unreacted MoSi_2_ and face-centered Al were found in the final product. In samples S1 and S2, no pure Al was detected, but the Si-saturated Al-rich Al_0.85_Si_0.15_ phase was detected. Under the chosen conditions (100.8–134.4 J·mm^−3^ ED) and with the addition of 30 wt.% AlSi10Mg to the MoSi_2_, the C11b tetragonal lattice of MoSi_2_ expanded until achieving a hexagonal Mo(Si_0.67_Al_0.33_)_2_ (x = 0.33 mol) structure, while no further C40 lattice expansion was detected in forming the Al-rich orthorhombic C54 structure. In contrast, the formation of C54 orthorhombic structures with Mo_10_Si_7_Al_13_, MoAl_1.3_Si_0.7_, and Al_4_Mo_3_Si_2_ is claimed in [4,9,10,11]; the formation of the silicon lean Al_8_Mo_3_ phase is reported in [14].

**Figure 10:**
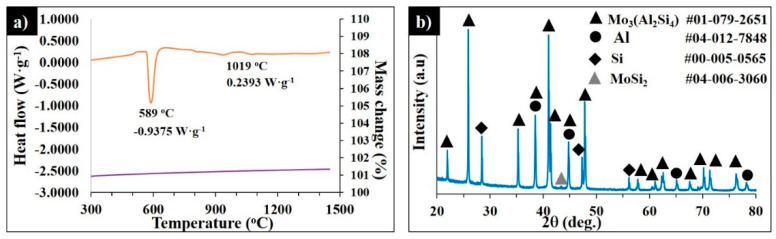
DSC-TG curves of MoSi_2_-30 wt.% AlSi10Mg heated up to 1450 °C at 20°·min^−1^ in Ar (**a**) and the corresponding XRD pattern of the quenched sample (**b**).

To study the mechanism of the MoSi_2_–AlSi10Mg interaction, the thermogravimetric analysis for MoSi_2_-30 wt.% AlSi10Mg was performed. Figure 10a shows the DSC-TG curves of the MoSi_2_-30 wt.% AlSi10Mg mixture heated up to 1450 °C. The DSC curves exhibit the endothermic peak of the reaction of the aluminum melting and the Al-Si eutectic phase formation, where the heat absorption recorded was 0.9575W·g^−1^. The weak exothermic reaction was observed starting from 944 °C and the maximum heat flow was 0.2393 W·g^−1^ at 1019 °C.

No further chemical reactions were detected. According to the XRD patterns in Figure 10b, the sample contained 62.4% Mo_3_(Al_2_Si_4_), 0.7% MoSi_2_, 12.4% Si, 3.3% MoAl_0.6_Si_1.4_, and 21.2% Al; however, the Al_0.85_Si_0.15_ phase was not detected.

The measured hardness of all SLM-processed materials was HV1 ~ 545 ± 48, which was around four times higher than for the printed AlSi10Mg alloy [21]. The high hardness is attributed to the significant effect of the MoSi_2_ addition and the development of new phases during solidification.

## 4. Conclusions

Nowadays, there is huge interest in the development of new alloys for AM processes. The in situ SLM of mixed powders allows the production of hard materials with tailored properties, taking into consideration specific needs.

Fully dense Mo(Si_1-x_,Al_x_)_2_-based composites with hardness exceeding 500 HV1 were successfully SLM-processed at laser volumetric energy density values of 100.8 and 134.4 J·mm^−3^ and with scanning speeds of either 500 or 1000 mm s^−1^ from a MoSi_2_-30 wt.% AlSi10Mg powder mixture. The bulks demonstrated relatively low roughness for both the top (horizontal) and side (vertical) surfaces, indicating the good quality of the produced parts.

Under the chosen conditions, with the addition of 30 wt.% AlSi10Mg to the MoSi_2_, the C11b tetragonal lattice of MoSi_2_ was expanded until achieving a hexagonal Mo(Si_0.67_Al_0.33_)_2_ (x = 0.33 mol) structure, while no further C40 lattice expansion was detected in forming the Al-rich orthorhombic C54 structure.

## Figures and Tables

**Table 1:** Methods of Mo(Si,Al)_2_ fabrication.

Initial Reagents	Preparation Technique	Final Product	Important Notes	Refs.
Mo + 2(1 − x)Si + 2xAl x *=* 0.14; 0.15; 0.28; 0.37	Arc melting of plates of the constituent elements Mo, Si, and Al in argon.	Mo(Si_1-*x*,_Al*_x_ x*)_2_ (0.11 < x *<* 0.55) x *=* 0.14; 0.15; 0.28; 0.37	The substitution of Si with Al gives Mo(Si_1-x_,Al_x_)_2_ of the C40 structure with a large homogeneity in the range of 0.11 < x < 0.55	[3]
33.3 at.% Mo + 44.2 at.% Si + 22.5 at.% Al	Arc melting	C40 Mo(Al_0. 5_,Si_0,5_)_2_ and C54 MoAl_1.3_Si_0.7_ phases	Addition of Al as a substitution for Si leads to formation of the higher symmetry C40 and C54 phases	[4]
Mo + 2(1 − x)Si + 2xAl x = 0.0075–0.225	Arc melting	Mo(Si_0.9925_Al_0.0075)2_ Mo(Si_0.985_Al_0.015)2_ Mo(Si_0.925_Al_0.075)2_ Mo(Si_0.85_Al_0.15)2_ Mo(Si_0.775_Al_0.225)2_	Al is soluble in MoSi_2_ up to about x = 0.045. The excess of Al resulted in formation of the C40- or C49-type phases	[5]
Mo + 2Si + (1–2.5 at.%)Al	Arc melting	Mo(Al, Si)_2_ Al-(1–2.5 at.%)	The addition of 2 at.% Al increased the high-temperature strength of Mo(Al, Si)_2_, lowered the brittle to ductile transition temperature, and decreased the hardness	[6]
Mo + 2(1 − x)Si + 2xAl x = 0.01–0.5	Self-propagating high-temperature synthesis (SHS) + hot pressing	Mo(Al_0.5_,Si_0.5_)_2_ Mo(Al_0.2_,Si_0.8_)_2_ Mo(Al_0.05_,Si_0.95_)_2_ Mo(Al_0.01_,Si_0.99_)_2_	The substitution of 10 wt.% Al for Si yielded equal amounts of Mo(Al,Si)_2_ and MoSi_2_	[7]
Mo-2Si-0.04PTFE-0.88Al Mo-2Si-0.08PTFE-0.88Al	SHS	MoSi_2_, Al, Mo_5_Si_3_, AlF_3_ in MoSi_2_-Al cermet foam	Porous product	[8]
Mo + 2(1 − x) + 2x x = 0–0.5	SHS + induction plasma spheroidization (IPS)	Mo(Si_1−*x*_,Al*_x_*)_2_ (x = 0–0.5) x = 0 → nearly pure C11b MoSi_2_ x = 0.1 → C11b C40 0.2 ≤ x ≤ 0.4 → C40 + C11b (trace) x = 0.1 → C40 + C54	Mo(Si,Al)_2_ with C40 structure designed as Mo(Si_0.6_,Al_0.4_)_2_ with the maximum Al content in SHS. After IPS, the apparent density was remarkable improved	[9]
Mo + 2(1 − x)Si + 2xAl x = 0.0–0.5 mole (0 to 17.86 wt.% Al)	SHS	Mo(Si_1−*x*_,Al*_x_*)_2_ x = 0.08 → C11b MoSi_2_ + C40 x = 0.2 → nearly pure C40 x = 0.5 → C40 + C54	Up to 2.84 wt.% Al, only C11b’s tetragonal phase is present; up to 5.33 wt.% Al, a duplex of C11b/C40 phases is present. Increasing the Al reduces the amount of C11b in the biphasic region. A single C40 hexagonal forms at 7.11 wt.% Al. At 17.86 wt.% Al, orthorhombic C54 (Al_4_Mo_3_Si_2_) appears	[10]
Mo + 2(1 − x) + 2x x = 0–0.6	Pseudo-HIP	Mo(Si_1-x_,Al_x_)_2_ x = 0 → C11b x = 0.1 → C11b + C40 x = 0.2−0.4 → C40 x = 0.5 → C40 + C54 x = 0.6 → C54	C40 is in the range of x = 0.1−0.5, while C11b is detected at x = 0.1 and C54 at x = 0.5. C11b is identified only atx = 0, while only C54 is detected at x = 0.6	[11]
SHS-ed powders Mo_1-x_Nb_x_)Si_2_ (x = 0–0.3), Mo(Si_1-y_,Al_y_)_2_ (y = 0–0.3)	SPS at 1350 °C and 40 MPa for 6 min in vacuum	(Mo_1-x_Nb_x_)Si_2_ Mo(Si_1-y_,Al_y_)_2_	Addition of Nb at x = 0–0.12 increased the strength and toughness	[12]
Mo plate and molten Al saturated with Si and Mo	Dipping Mo into Al-Si bath at 973 K for 350 ks	Mo plate covered with the layer of Mo(Si,Al)_2_ of Mo:Si:Al = 30:12:58	The needle-like grains grow perpendicular to Mo’s surface. The reaction goes through the solution–precipitation process in the Al(Si) liquid between Mo and Mo(Si,Al)_2_ layers	[13]

**Table 2:** Process parameters for samples S1–S3.

Sample ID	Laser Power (W)	Scanning Speed (mm·s^−1^)	Laser Volumetric Energy Density (J·mm^−3^)	Build Rate (mm^3^·s^−1^)
S1	150	500	100.8	1.48
S2	200	500	134.4	1.48
S3	300	1000	100.8	2.97

**Table 3:** Size distributions of the AlSi10Mg, MoSi_2_, and MoSi_2_-30 wt.% AlSi10Mg powders.

	Particle Size	D10 (µm)	D50 (µm)	D90 (µm)
Composition				
AlSiMg10		25.14 ± 0.53	38.72 ± 0.67	55.47 ± 0.44
MoSi_2_		5.44 ± 0.26	18.95 ± 0.89	43.45 ± 0.65
MoSi_2_-30 wt.% AlSi10Mg		7.68 ± 0.07	33.02 ± 1.18	54.19 ± 1.30

**Table 4:** Average flow rate for the MoSi_2_, MoSi_2_-30 wt.% AlSi10Mg, and AlSi10Mg alloy powders.

	Powders	MoSi_2_ (<45 µm)	MoSi_2_-30 wt.% AlSi10Mg	AlSi10Mg (15–63 µm)
FT4 Results				
BFE, mJ		307.52 ± 13.74	252.27 ± 19.41	206.14 ± 8.92
SI		1.09999 ± 0.00606	0.95012 ±0.07664	0.96271 ± 0.06536

**Table 5:** The roughness of the top and side surfaces of the printed samples.

Sample	Top Surface Roughness, µm		Side Surface Roughness, µm	
	Sa	Sz	Sa	Sz
S1	10.1	94.4	11.2	109.5
S2	9.9	75.5	13.2	130.8
S3	14.7	130.2	17.8	173.5

**Table 6:** Compositions of the samples estimated from XRD patterns via the Rietveld refinement method.

Sample ID	Composition (%)						Elemental Composition (%)		
	Mo_3_(Si_4_Al_2_)	MoAl_0.6_Si_1.4_	MoSi_2_	Al_0.85_Si_0.15_	Al	Si	Mo	Si	Al
S1	69.4	0.5	1.2	19.7	0.0	9.1	45.1	30.0	24.9
S2	70.9	0.7	1.1	17.5	0.0	9.7	46.1	30.6	23.3
S3	67.0	3.7	2.4	17.0	1.2	8.7	46.3	29.8	23.9
